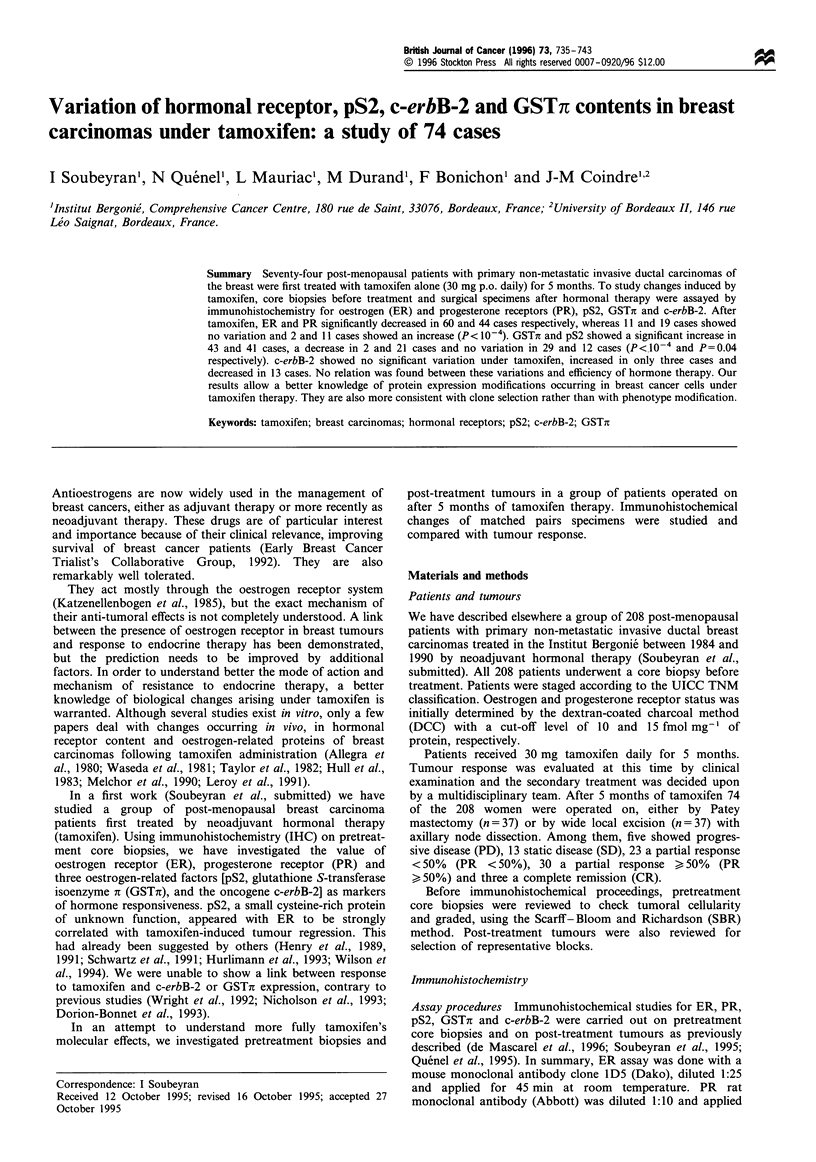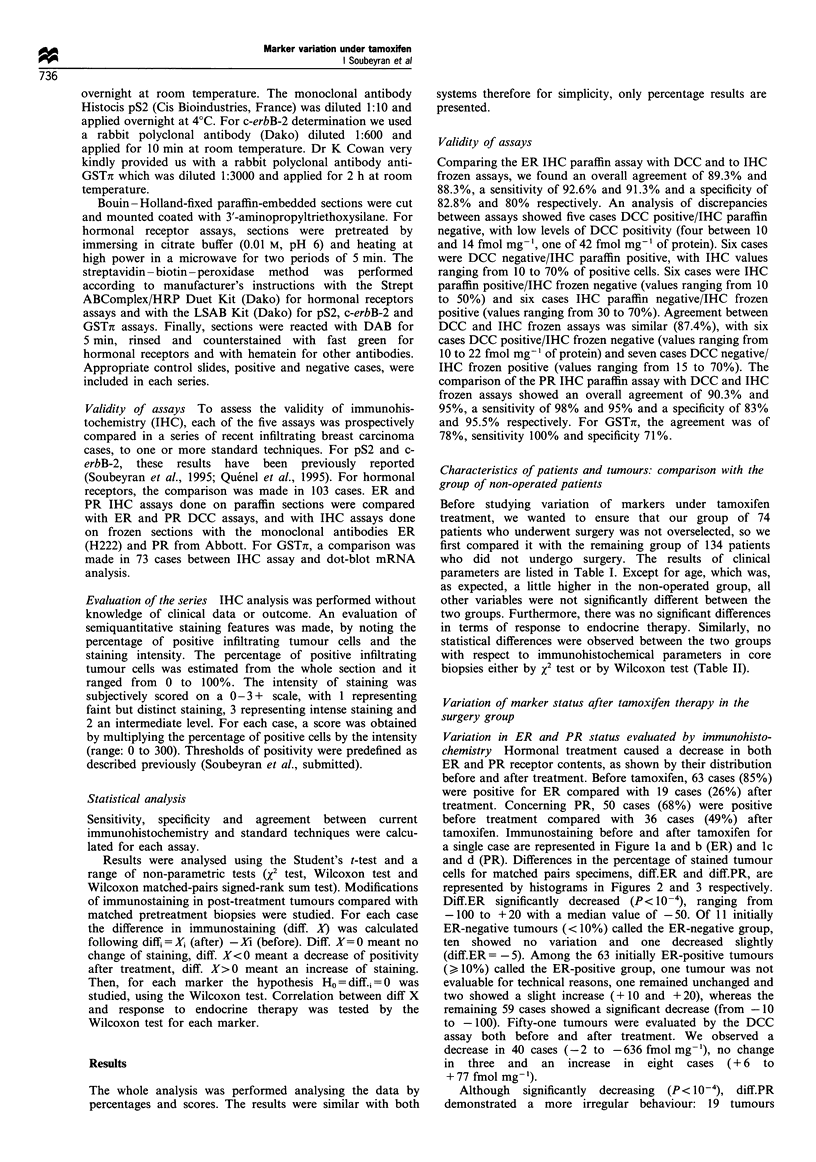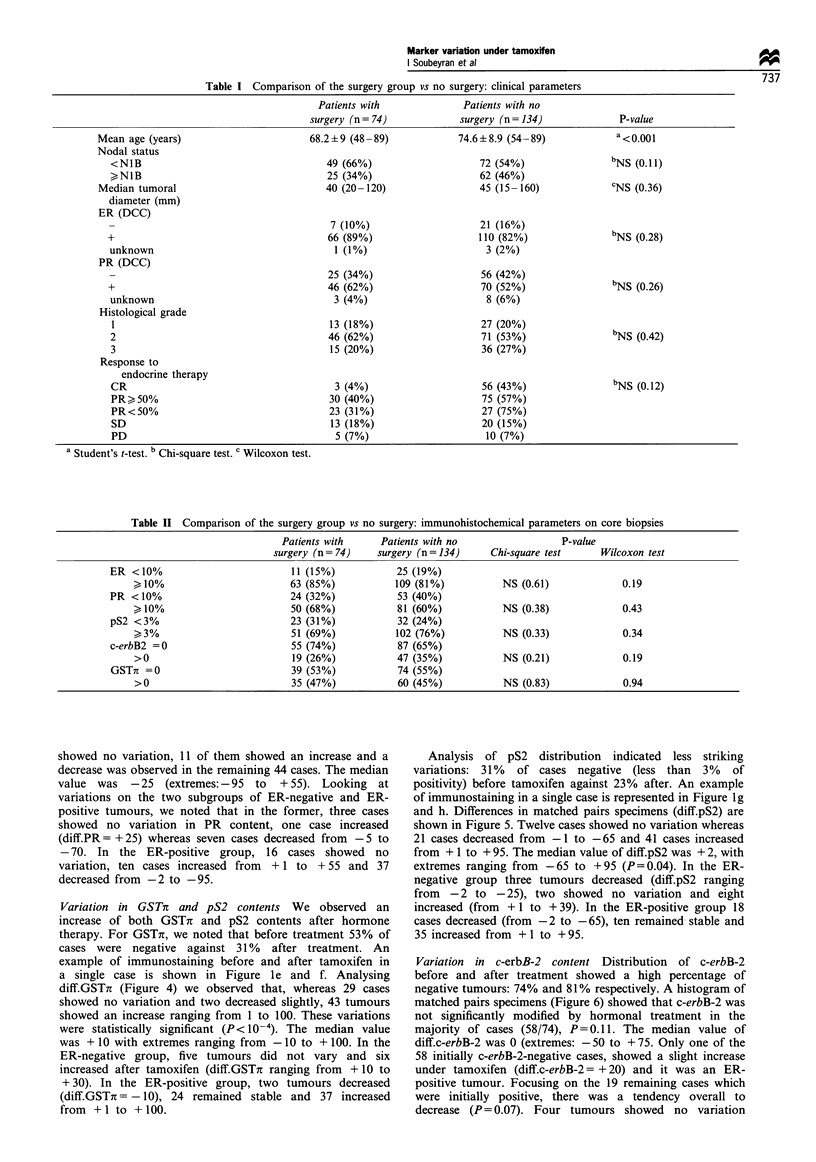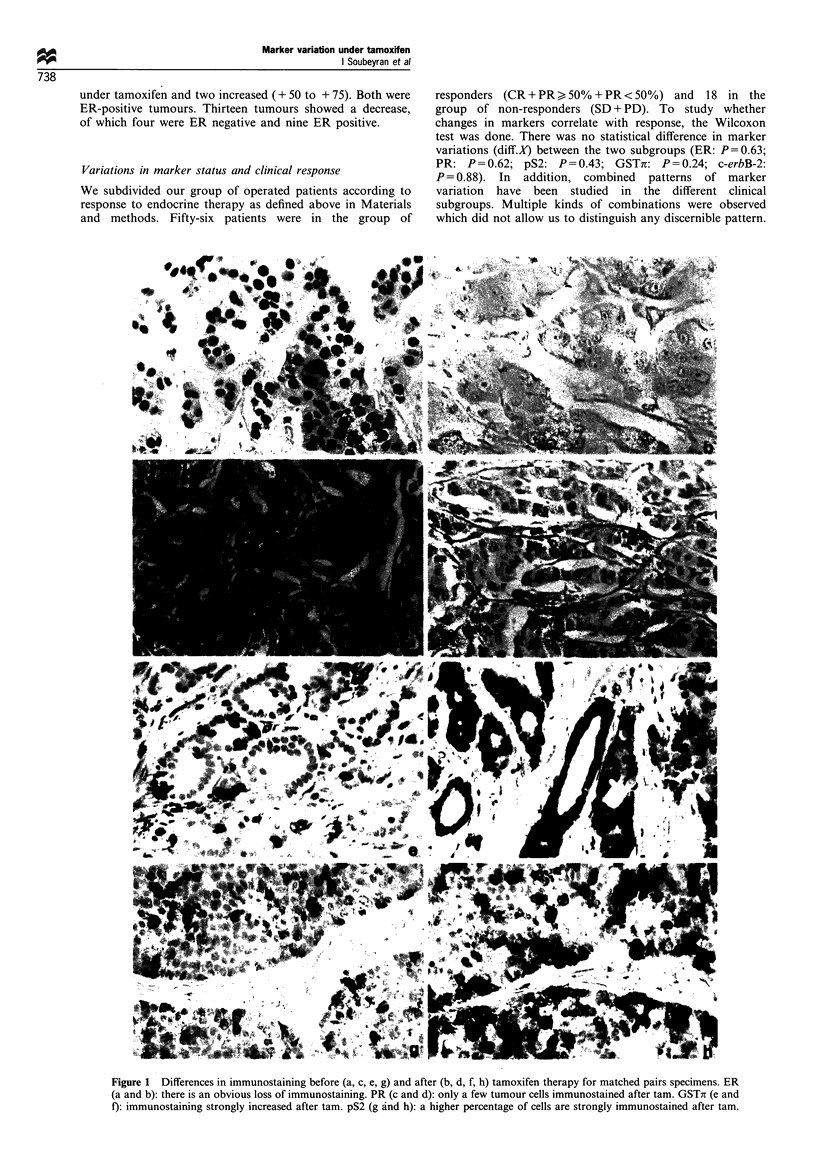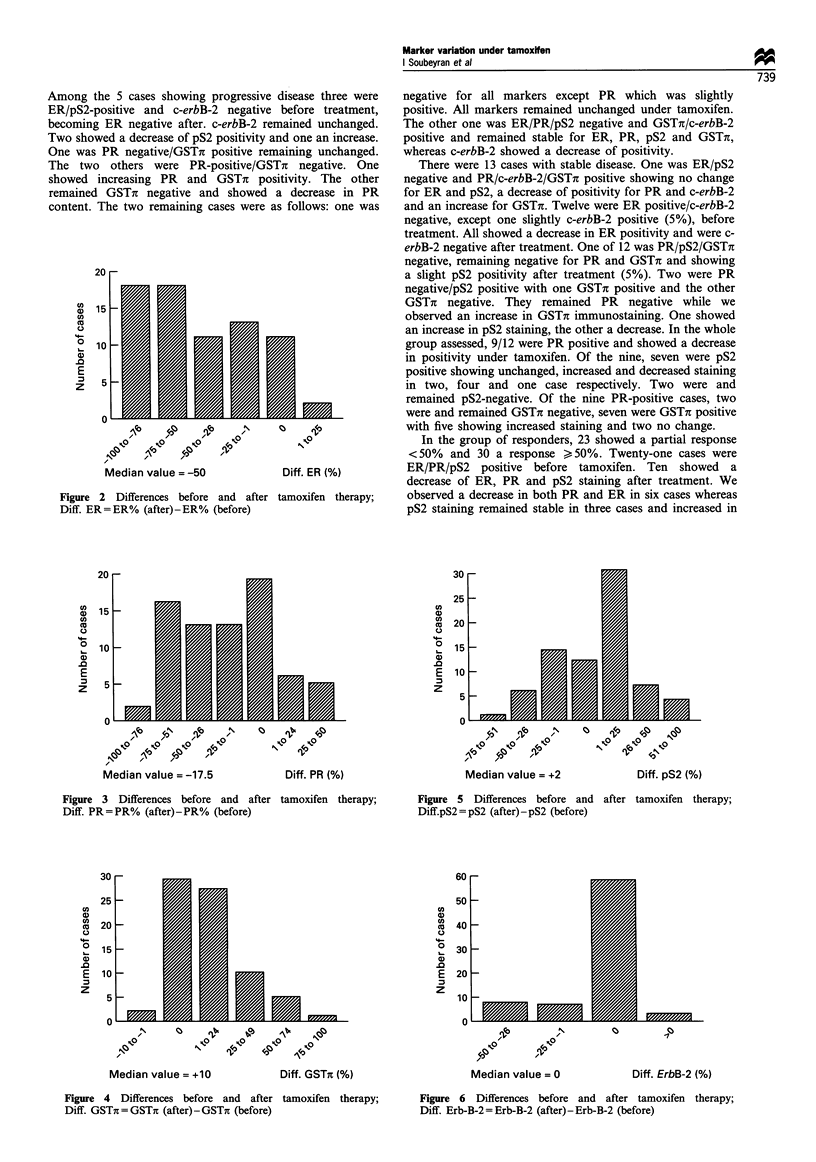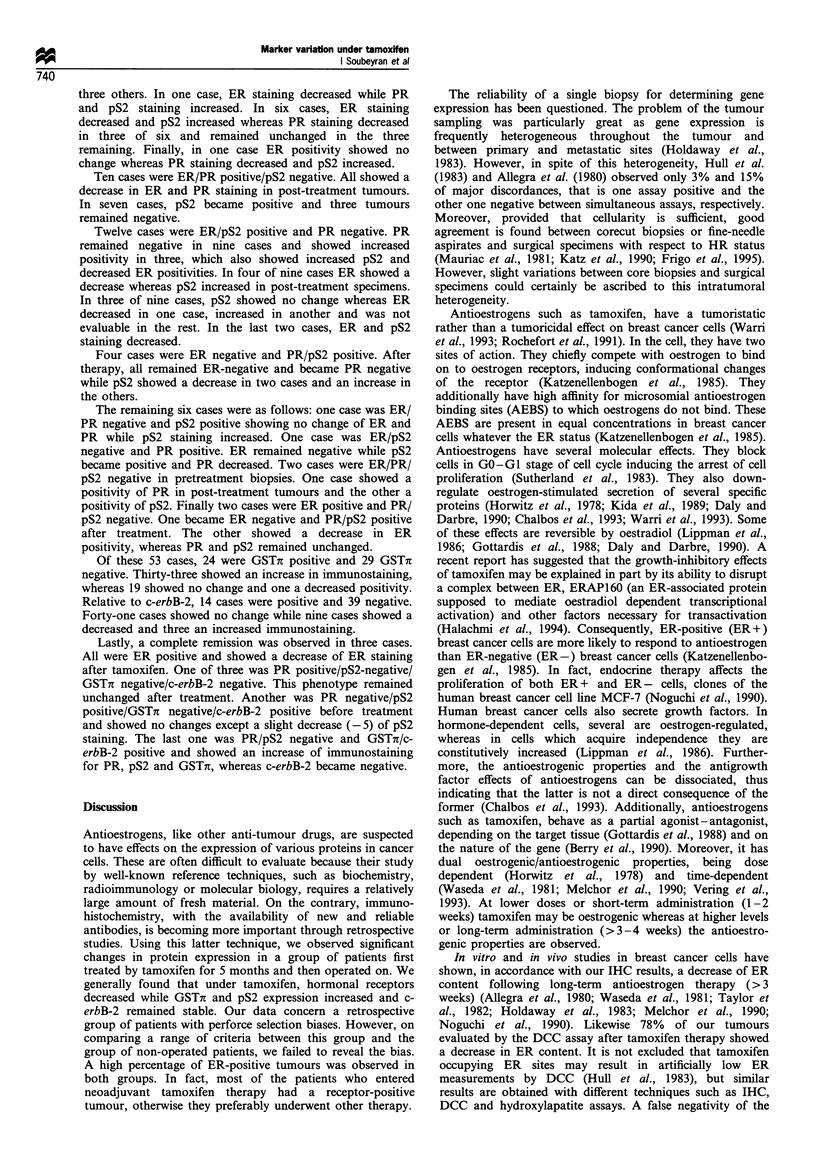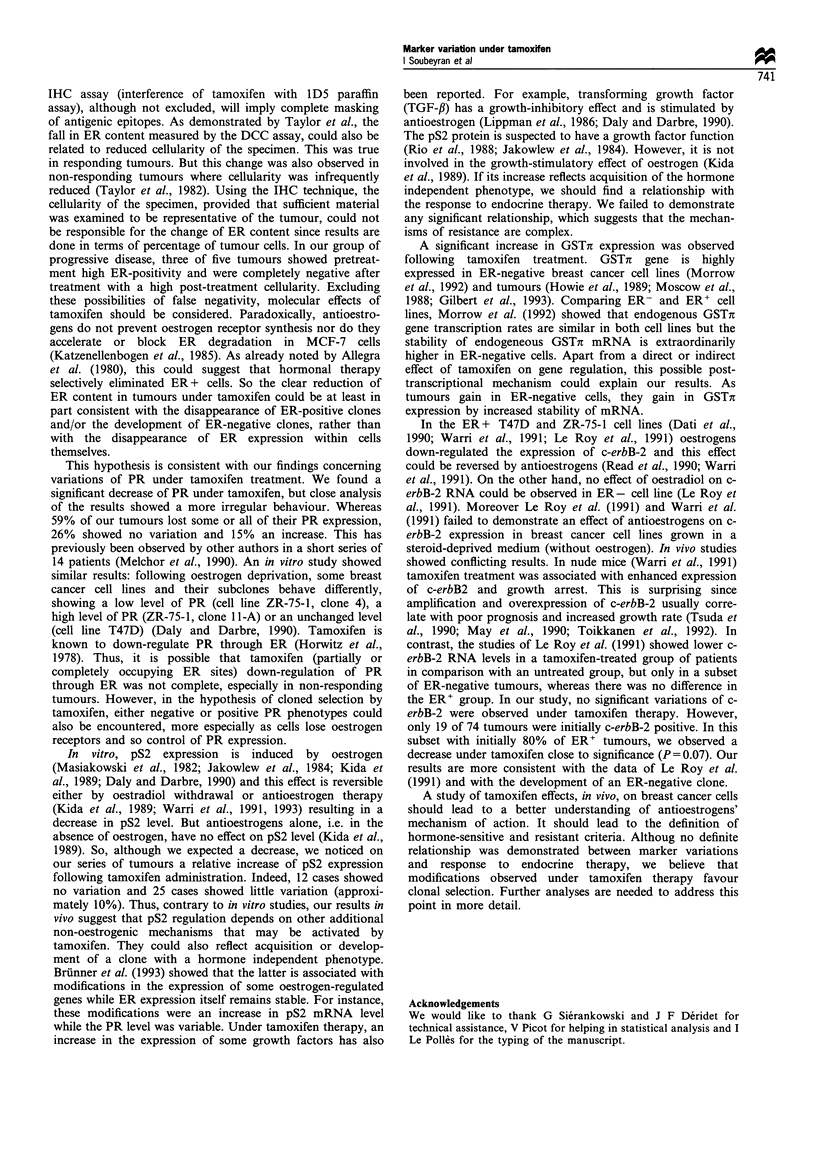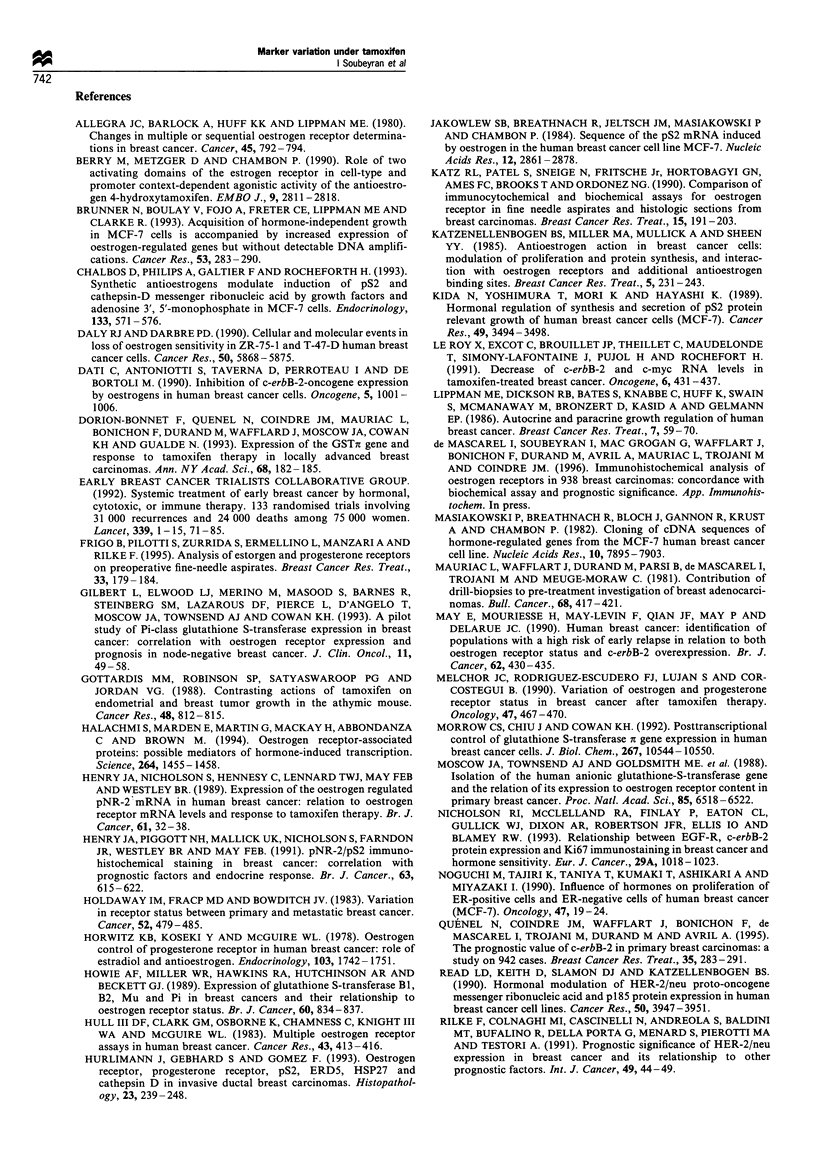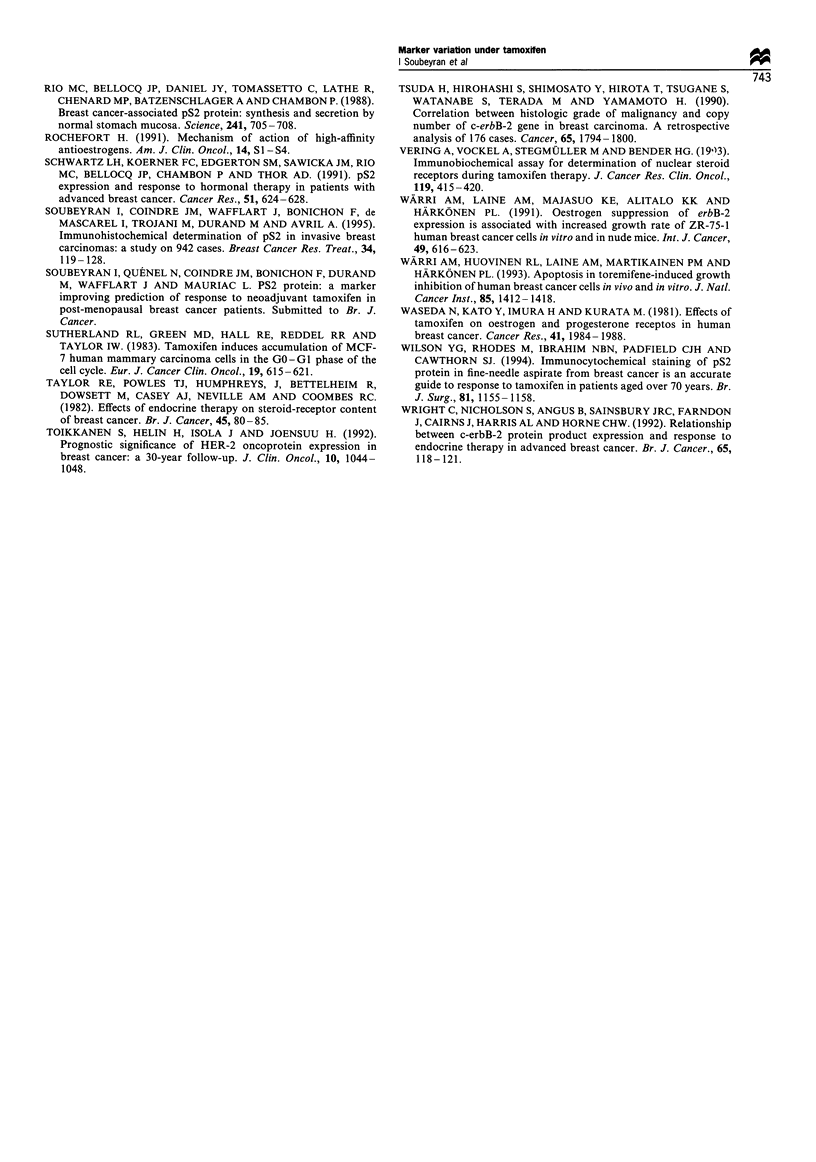# Variation of hormonal receptor, pS2, c-erbB-2 and GSTpi contents in breast carcinomas under tamoxifen: a study of 74 cases.

**DOI:** 10.1038/bjc.1996.129

**Published:** 1996-03

**Authors:** I. Soubeyran, N. Quénel, L. Mauriac, M. Durand, F. Bonichon

**Affiliations:** Institut Bergonie, Comprehensive Cancer Centre, Bordeaux, France.

## Abstract

**Images:**


British Journal of Cancer (1996) 73, 735-743
© 1996 Stockton Press All rights reserved 0007-0920/96 $12.00

### Variation of hormonal receptor, pS2, c-*erb*B-2 and GST$\pi$ contents in breast carcinomas under tamoxifen: a study of 74 cases

I Soubeyran[1], N Quénel[1], L Mauriac[1], M Durand[1], F Bonichon[1] and J-M Coindre[1,2]

[1]*Institut Bergonié, Comprehensive Cancer Centre, 180 rue de Saint, 33076, Bordeaux, France;* [2]*University of Bordeaux II, 146 rue Léo Saignat, Bordeaux, France.*

**Summary** Seventy-four post-menopausal patients with primary non-metastatic invasive ductal carcinomas of the breast were first treated with tamoxifen alone (30 mg p.o. daily) for 5 months. To study changes induced by tamoxifen, core biopsies before treatment and surgical specimens after hormonal therapy were assayed by immunohistochemistry for oestrogen (ER) and progesterone receptors (PR), pS2, GST$\pi$ and c-*erb*B-2. After tamoxifen, ER and PR significantly decreased in 60 and 44 cases respectively, whereas 11 and 19 cases showed no variation and 2 and 11 cases showed an increase ($P<10^{-4}$). GST$\pi$ and pS2 showed a significant increase in 43 and 41 cases, a decrease in 2 and 21 cases and no variation in 29 and 12 cases ($P<.10^{-4}$ and $P=0.04$ respectively). c-*erb*B-2 showed no significant variation under tamoxifen, increased in only three cases and decreased in 13 cases. No relation was found between these variations and efficiency of hormone therapy. Our results allow a better knowledge of protein expression modifications occurring in breast cancer cells under tamoxifen therapy. They are also more consistent with clone selection rather than with phenotype modification.

**Keywords:** tamoxifen; breast carcinomas; hormonal receptors; pS2; c-*erb*B-2; GST$\pi$

Antioestrogens are now widely used in the management of breast cancers, either as adjuvant therapy or more recently as neoadjuvant therapy. These drugs are of particular interest and importance because of their clinical relevance, improving survival of breast cancer patients (Early Breast Cancer Trialist's Collaborative Group, 1992). They are also remarkably well tolerated.

They act mostly through the oestrogen receptor system (Katzenellenbogen *et al.*, 1985), but the exact mechanism of their anti-tumoral effects is not completely understood. A link between the presence of oestrogen receptor in breast tumours and response to endocrine therapy has been demonstrated, but the prediction needs to be improved by additional factors. In order to understand better the mode of action and mechanism of resistance to endocrine therapy, a better knowledge of biological changes arising under tamoxifen is warranted. Although several studies exist *in vitro*, only a few papers deal with changes occurring *in vivo*, in hormonal receptor content and oestrogen-related proteins of breast carcinomas following tamoxifen administration (Allegra *et al.*, 1980; Waseda *et al.*, 1981; Taylor *et al.*, 1982; Hull *et al.*, 1983; Melchor *et al.*, 1990; Leroy *et al.*, 1991).

In a first work (Soubeyran *et al.*, submitted) we have studied a group of post-menopausal breast carcinoma patients first treated by neoadjuvant hormonal therapy (tamoxifen). Using immunohistochemistry (IHC) on pretreatment core biopsies, we have investigated the value of oestrogen receptor (ER), progesterone receptor (PR) and three oestrogen-related factors [pS2, glutathione *S*-transferase isoenzyme $\pi$ (GST$\pi$), and the oncogene c-*erb*B-2] as markers of hormone responsiveness. pS2, a small cysteine-rich protein of unknown function, appeared with ER to be strongly correlated with tamoxifen-induced tumour regression. This had already been suggested by others (Henry *et al.*, 1989, 1991; Schwartz *et al.*, 1991; Hurlimann *et al.*, 1993; Wilson *et al.*, 1994). We were unable to show a link between response to tamoxifen and c-*erb*B-2 or GST$\pi$ expression, contrary to previous studies (Wright *et al.*, 1992; Nicholson *et al.*, 1993; Dorion-Bonnet *et al.*, 1993).

In an attempt to understand more fully tamoxifen's molecular effects, we investigated pretreatment biopsies and post-treatment tumours in a group of patients operated on after 5 months of tamoxifen therapy. Immunohistochemical changes of matched pairs specimens were studied and compared with tumour response.

### Materials and methods

### Patients and tumours

We have described elsewhere a group of 208 post-menopausal patients with primary non-metastatic invasive ductal breast carcinomas treated in the Institut Bergonié between 1984 and 1990 by neoadjuvant hormonal therapy (Soubeyran *et al.*, submitted). All 208 patients underwent a core biopsy before treatment. Patients were staged according to the UICC TNM classification. Oestrogen and progesterone receptor status was initially determined by the dextran-coated charcoal method (DCC) with a cut-off level of 10 and 15 fmol mg$^{-1}$ of protein, respectively.

Patients received 30 mg tamoxifen daily for 5 months. Tumour response was evaluated at this time by clinical examination and the secondary treatment was decided upon by a multidisciplinary team. After 5 months of tamoxifen 74 of the 208 women were operated on, either by Patey mastectomy ($n=37$) or by wide local excision ($n=37$) with axillary node dissection. Among them, five showed progressive disease (PD), 13 static disease (SD), 23 a partial response <50% (PR <50%), 30 a partial response ⩾50% (PR ⩾50%) and three a complete remission (CR).

Before immunohistochemical proceedings, pretreatment core biopsies were reviewed to check tumoral cellularity and graded, using the Scarff-Bloom and Richardson (SBR) method. Post-treatment tumours were also reviewed for selection of representative blocks.

### Immunohistochemistry

*Assay procedures* Immunohistochemical studies for ER, PR, pS2, GST$\pi$ and c-*erb*B-2 were carried out on pretreatment core biopsies and on post-treatment tumours as previously described (de Mascarel *et al.*, 1996; Soubeyran *et al.*, 1995; Quénel *et al.*, 1995). In summary, ER assay was done with a mouse monoclonal antibody clone 1D5 (Dako), diluted 1:25 and applied for 45 min at room temperature. PR rat monoclonal antibody (Abbott) was diluted 1:10 and applied

Correspondence: I Soubeyran
Received 12 October 1995; revised 16 October 1995; accepted 27 October 1995

overnight at room temperature. The monoclonal antibody Histocis pS2 (Cis Bioindustries, France) was diluted 1:10 and applied overnight at 4°C. For c-*erb*B-2 determination we used a rabbit polyclonal antibody (Dako) diluted 1:600 and applied for 10 min at room temperature. Dr K Cowan very kindly provided us with a rabbit polyclonal antibody anti-GST$\pi$ which was diluted 1:3000 and applied for 2 h at room temperature.

Bouin–Holland-fixed paraffin-embedded sections were cut and mounted coated with 3′-aminopropyltriethoxysilane. For hormonal receptor assays, sections were pretreated by immersing in citrate buffer (0.01 M, pH 6) and heating at high power in a microwave for two periods of 5 min. The streptavidin–biotin–peroxidase method was performed according to manufacturer's instructions with the Strept ABComplex/HRP Duet Kit (Dako) for hormonal receptors assays and with the LSAB Kit (Dako) for pS2, c-*erb*B-2 and GST$\pi$ assays. Finally, sections were reacted with DAB for 5 min, rinsed and counterstained with fast green for hormonal receptors and with hematein for other antibodies. Appropriate control slides, positive and negative cases, were included in each series.

*Validity of assays* To assess the validity of immunohistochemistry (IHC), each of the five assays was prospectively compared in a series of recent infiltrating breast carcinoma cases, to one or more standard techniques. For pS2 and c-*erb*B-2, these results have been previously reported (Soubeyran *et al.*, 1995; Quénel *et al.*, 1995). For hormonal receptors, the comparison was made in 103 cases. ER and PR IHC assays done on paraffin sections were compared with ER and PR DCC assays, and with IHC assays done on frozen sections with the monoclonal antibodies ER (H222) and PR from Abbott. For GST$\pi$, a comparison was made in 73 cases between IHC assay and dot-blot mRNA analysis.

*Evaluation of the series* IHC analysis was performed without knowledge of clinical data or outcome. An evaluation of semiquantitative staining features was made, by noting the percentage of positive infiltrating tumour cells and the staining intensity. The percentage of positive infiltrating tumour cells was estimated from the whole section and it ranged from 0 to 100%. The intensity of staining was subjectively scored on a 0–3+ scale, with 1 representing faint but distinct staining, 3 representing intense staining and 2 an intermediate level. For each case, a score was obtained by multiplying the percentage of positive cells by the intensity (range: 0 to 300). Thresholds of positivity were predefined as described previously (Soubeyran *et al.*, submitted).

### *Statistical analysis*

Sensitivity, specificity and agreement between current immunohistochemistry and standard techniques were calculated for each assay.

Results were analysed using the Student's *t*-test and a range of non-parametric tests ($\chi^2$ test, Wilcoxon test and Wilcoxon matched-pairs signed-rank sum test). Modifications of immunostaining in post-treatment tumours compared with matched pretreatment biopsies were studied. For each case the difference in immunostaining (diff. $X$) was calculated following $\text{diff}_i = X_i$ (after) $- X\text{i}$ (before). Diff. $X=0$ meant no change of staining, diff. $X<0$ meant a decrease of positivity after treatment, diff. $X>0$ meant an increase of staining. Then, for each marker the hypothesis $H_0 = \text{diff.}_i = 0$ was studied, using the Wilcoxon test. Correlation between diff X and response to endocrine therapy was tested by the Wilcoxon test for each marker.

### Results

The whole analysis was performed analysing the data by percentages and scores. The results were similar with both systems therefore for simplicity, only percentage results are presented.

### *Validity of assays*

Comparing the ER IHC paraffin assay with DCC and to IHC frozen assays, we found an overall agreement of 89.3% and 88.3%, a sensitivity of 92.6% and 91.3% and a specificity of 82.8% and 80% respectively. An analysis of discrepancies between assays showed five cases DCC positive/IHC paraffin negative, with low levels of DCC positivity (four between 10 and 14 fmol mg$^{-1}$, one of 42 fmol mg$^{-1}$ of protein). Six cases were DCC negative/IHC paraffin positive, with IHC values ranging from 10 to 70% of positive cells. Six cases were IHC paraffin positive/IHC frozen negative (values ranging from 10 to 50%) and six cases IHC paraffin negative/IHC frozen positive (values ranging from 30 to 70%). Agreement between DCC and IHC frozen assays was similar (87.4%), with six cases DCC positive/IHC frozen negative (values ranging from 10 to 22 fmol mg$^{-1}$ of protein) and seven cases DCC negative/IHC frozen positive (values ranging from 15 to 70%). The comparison of the PR IHC paraffin assay with DCC and IHC frozen assays showed an overall agreement of 90.3% and 95%, a sensitivity of 98% and 95% and a specificity of 83% and 95.5% respectively. For GST$\pi$, the agreement was of 78%, sensitivity 100% and specificity 71%.

### *Characteristics of patients and tumours: comparison with the group of non-operated patients*

Before studying variation of markers under tamoxifen treatment, we wanted to ensure that our group of 74 patients who underwent surgery was not overselected, so we first compared it with the remaining group of 134 patients who did not undergo surgery. The results of clinical parameters are listed in Table I. Except for age, which was, as expected, a little higher in the non-operated group, all other variables were not significantly different between the two groups. Furthermore, there was no significant differences in terms of response to endocrine therapy. Similarly, no statistical differences were observed between the two groups with respect to immunohistochemical parameters in core biopsies either by $\chi^2$ test or by Wilcoxon test (Table II).

### *Variation of marker status after tamoxifen therapy in the surgery group*

*Variation in ER and PR status evaluated by immunohistochemistry* Hormonal treatment caused a decrease in both ER and PR receptor contents, as shown by their distribution before and after treatment. Before tamoxifen, 63 cases (85%) were positive for ER compared with 19 cases (26%) after treatment. Concerning PR, 50 cases (68%) were positive before treatment compared with 36 cases (49%) after tamoxifen. Immunostaining before and after tamoxifen for a single case are represented in Figure 1a and b (ER) and 1c and d (PR). Differences in the percentage of stained tumour cells for matched pairs specimens, diff.ER and diff.PR, are represented by histograms in Figures 2 and 3 respectively. Diff.ER significantly decreased ($P<10^{-4}$), ranging from $-100$ to $+20$ with a median value of $-50$. Of 11 initially ER-negative tumours (<10%) called the ER-negative group, ten showed no variation and one decreased slightly (diff.ER = $-5$). Among the 63 initially ER-positive tumours ($\geqslant$10%) called the ER-positive group, one tumour was not evaluable for technical reasons, one remained unchanged and two showed a slight increase (+10 and +20), whereas the remaining 59 cases showed a significant decrease (from $-10$ to $-100$). Fifty-one tumours were evaluated by the DCC assay both before and after treatment. We observed a decrease in 40 cases ($-2$ to $-636$ fmol mg$^{-1}$), no change in three and an increase in eight cases (+6 to +77 fmol mg$^{-1}$).

Although significantly decreasing ($P<10^{-4}$), diff.PR demonstrated a more irregular behaviour: 19 tumours

**Table I** Comparison of the surgery group *vs* no surgery: clinical parameters

| | *Patients with surgery (n=74)* | *Patients with no surgery (n=134)* | P-*value* |
|---|---|---|---|
| Mean age (years) | 68.2±9 (48–89) | 74.6±8.9 (54–89) | [a]<0.001 |
| Nodal status | | | |
| <N1B | 49 (66%) | 72 (54%) | [b]NS (0.11) |
| ⩾N1B | 25 (34%) | 62 (46%) | |
| Median tumoral diameter (mm) | 40 (20–120) | 45 (15–160) | [c]NS (0.36) |
| ER (DCC) | | | |
| – | 7 (10%) | 21 (16%) | |
| + | 66 (89%) | 110 (82%) | [b]NS (0.28) |
| unknown | 1 (1%) | 3 (2%) | |
| PR (DCC) | | | |
| – | 25 (34%) | 56 (42%) | |
| + | 46 (62%) | 70 (52%) | [b]NS (0.26) |
| unknown | 3 (4%) | 8 (6%) | |
| Histological grade | | | |
| 1 | 13 (18%) | 27 (20%) | |
| 2 | 46 (62%) | 71 (53%) | [b]NS (0.42) |
| 3 | 15 (20%) | 36 (27%) | |
| Response to endocrine therapy | | | |
| CR | 3 (4%) | 56 (43%) | [b]NS (0.12) |
| PR⩾50% | 30 (40%) | 75 (57%) | |
| PR<50% | 23 (31%) | 27 (75%) | |
| SD | 13 (18%) | 20 (15%) | |
| PD | 5 (7%) | 10 (7%) | |

[a] Student's *t*-test. [b] Chi-square test. [c] Wilcoxon test.

**Table II** Comparison of the surgery group *vs* no surgery: immunohistochemical parameters on core biopsies

| | *Patients with surgery (n=74)* | *Patients with no surgery (n=134)* | P-*value* Chi-square test | *Wilcoxon test* |
|---|---|---|---|---|
| ER <10% | 11 (15%) | 25 (19%) | | |
| ⩾10% | 63 (85%) | 109 (81%) | NS (0.61) | 0.19 |
| PR <10% | 24 (32%) | 53 (40%) | | |
| ⩾10% | 50 (68%) | 81 (60%) | NS (0.38) | 0.43 |
| pS2 <3% | 23 (31%) | 32 (24%) | | |
| ⩾3% | 51 (69%) | 102 (76%) | NS (0.33) | 0.34 |
| c-*erb*B2 =0 | 55 (74%) | 87 (65%) | | |
| >0 | 19 (26%) | 47 (35%) | NS (0.21) | 0.19 |
| GSTπ =0 | 39 (53%) | 74 (55%) | | |
| >0 | 35 (47%) | 60 (45%) | NS (0.83) | 0.94 |

showed no variation, 11 of them showed an increase and a decrease was observed in the remaining 44 cases. The median value was −25 (extremes: −95 to +55). Looking at variations on the two subgroups of ER-negative and ER-positive tumours, we noted that in the former, three cases showed no variation in PR content, one case increased (diff.PR= +25) whereas seven cases decreased from −5 to −70. In the ER-positive group, 16 cases showed no variation, ten cases increased from +1 to +55 and 37 decreased from −2 to −95.

*Variation in GSTπ and pS2 contents* We observed an increase of both GSTπ and pS2 contents after hormone therapy. For GSTπ, we noted that before treatment 53% of cases were negative against 31% after treatment. An example of immunostaining before and after tamoxifen in a single case is shown in Figure 1e and f. Analysing diff.GSTπ (Figure 4) we observed that, whereas 29 cases showed no variation and two decreased slightly, 43 tumours showed an increase ranging from 1 to 100. These variations were statistically significant ($P<10^{-4}$). The median value was +10 with extremes ranging from −10 to +100. In the ER-negative group, five tumours did not vary and six increased after tamoxifen (diff.GSTπ ranging from +10 to +30). In the ER-positive group, two tumours decreased (diff.GSTπ= −10), 24 remained stable and 37 increased from +1 to +100.

Analysis of pS2 distribution indicated less striking variations: 31% of cases negative (less than 3% of positivity) before tamoxifen against 23% after. An example of immunostaining in a single case is represented in Figure 1g and h. Differences in matched pairs specimens (diff.pS2) are shown in Figure 5. Twelve cases showed no variation whereas 21 cases decreased from −1 to −65 and 41 cases increased from +1 to +95. The median value of diff.pS2 was +2, with extremes ranging from −65 to +95 ($P=0.04$). In the ER-negative group three tumours decreased (diff.pS2 ranging from −2 to −25), two showed no variation and eight increased (from +1 to +39). In the ER-positive group 18 cases decreased (from −2 to −65), ten remained stable and 35 increased from +1 to +95.

*Variation in c-*erb*B-2 content* Distribution of c-*erb*B-2 before and after treatment showed a high percentage of negative tumours: 74% and 81% respectively. A histogram of matched pairs specimens (Figure 6) showed that c-*erb*B-2 was not significantly modified by hormonal treatment in the majority of cases (58/74), $P=0.11$. The median value of diff.c-*erb*B-2 was 0 (extremes: −50 to +75. Only one of the 58 initially c-*erb*B-2-negative cases, showed a slight increase under tamoxifen (diff.c-*erb*B-2= +20) and it was an ER-positive tumour. Focusing on the 19 remaining cases which were initially positive, there was a tendency overall to decrease ($P=0.07$). Four tumours showed no variation under tamoxifen and two increased (+50 to +75). Both were ER-positive tumours. Thirteen tumours showed a decrease, of which four were ER negative and nine ER positive.

*Variations in marker status and clinical response*

We subdivided our group of operated patients according to response to endocrine therapy as defined above in Materials and methods. Fifty-six patients were in the group of responders (CR+PR$\geqslant$50%+PR<50%) and 18 in the group of non-responders (SD+PD). To study whether changes in markers correlate with response, the Wilcoxon test was done. There was no statistical difference in marker variations (diff.$X$) between the two subgroups (ER: $P=0.63$; PR: $P=0.62$; pS2: $P=0.43$; GST$\pi$: $P=0.24$; c-*erb*B-2: $P=0.88$). In addition, combined patterns of marker variation have been studied in the different clinical subgroups. Multiple kinds of combinations were observed which did not allow us to distinguish any discernible pattern.

**Figure 1** Differences in immunostaining before (a, c, e, g) and after (b, d, f, h) tamoxifen therapy for matched pairs specimens. ER (a and b): there is an obvious loss of immunostaining. PR (c and d): only a few tumour cells immunostained after tam. GST$\pi$ (e and f): immunostaining strongly increased after tam. pS2 (g and h): a higher percentage of cells are strongly immunostained after tam.

Among the 5 cases showing progressive disease three were ER/pS2-positive and c-*erb*B-2 negative before treatment, becoming ER negative after. c-*erb*B-2 remained unchanged. Two showed a decrease of pS2 positivity and one an increase. One was PR negative/GST$\pi$ positive remaining unchanged. The two others were PR-positive/GST$\pi$ negative. One showed increasing PR and GST$\pi$ positivity. The other remained GST$\pi$ negative and showed a decrease in PR content. The two remaining cases were as follows: one was negative for all markers except PR which was slightly positive. All markers remained unchanged under tamoxifen. The other one was ER/PR/pS2 negative and GST$\pi$/c-*erb*B-2 positive and remained stable for ER, PR, pS2 and GST$\pi$, whereas c-*erb*B-2 showed a decrease of positivity.

There were 13 cases with stable disease. One was ER/pS2 negative and PR/c-*erb*B-2/GST$\pi$ positive showing no change for ER and pS2, a decrease of positivity for PR and c-*erb*B-2 and an increase for GST$\pi$. Twelve were ER positive/c-*erb*B-2 negative, except one slightly c-*erb*B-2 positive (5%), before treatment. All showed a decrease in ER positivity and were c-*erb*B-2 negative after treatment. One of 12 was PR/pS2/GST$\pi$ negative, remaining negative for PR and GST$\pi$ and showing a slight pS2 positivity after treatment (5%). Two were PR negative/pS2 positive with one GST$\pi$ positive and the other GST$\pi$ negative. They remained PR negative while we observed an increase in GST$\pi$ immunostaining. One showed an increase in pS2 staining, the other a decrease. In the whole group assessed, 9/12 were PR positive and showed a decrease in positivity under tamoxifen. Of the nine, seven were pS2 positive showing unchanged, increased and decreased staining in two, four and one case respectively. Two were and remained pS2-negative. Of the nine PR-positive cases, two were and remained GST$\pi$ negative, seven were GST$\pi$ positive with five showing increased staining and two no change.

In the group of responders, 23 showed a partial response <50% and 30 a response $\geqslant$50%. Twenty-one cases were ER/PR/pS2 positive before tamoxifen. Ten showed a decrease of ER, PR and pS2 staining after treatment. We observed a decrease in both PR and ER in six cases whereas pS2 staining remained stable in three cases and increased in

**Figure 2** Differences before and after tamoxifen therapy; Diff. ER = ER% (after) − ER% (before)

**Figure 3** Differences before and after tamoxifen therapy; Diff. PR = PR% (after) − PR% (before)

**Figure 5** Differences before and after tamoxifen therapy; Diff.pS2 = pS2 (after) − pS2 (before)

**Figure 4** Differences before and after tamoxifen therapy; Diff. GST$\pi$ = GST$\pi$ (after) − GST$\pi$ (before)

**Figure 6** Differences before and after tamoxifen therapy; Diff. Erb-B-2 = Erb-B-2 (after) − Erb-B-2 (before)

three others. In one case, ER staining decreased while PR and pS2 staining increased. In six cases, ER staining decreased and pS2 increased whereas PR staining decreased in three of six and remained unchanged in the three remaining. Finally, in one case ER positivity showed no change whereas PR staining decreased and pS2 increased.

Ten cases were ER/PR positive/pS2 negative. All showed a decrease in ER and PR staining in post-treatment tumours. In seven cases, pS2 became positive and three tumours remained negative.

Twelve cases were ER/pS2 positive and PR negative. PR remained negative in nine cases and showed increased positivity in three, which also showed increased pS2 and decreased ER positivities. In four of nine cases ER showed a decrease whereas pS2 increased in post-treatment specimens. In three of nine cases, pS2 showed no change whereas ER decreased in one case, increased in another and was not evaluable in the rest. In the last two cases, ER and pS2 staining decreased.

Four cases were ER negative and PR/pS2 positive. After therapy, all remained ER-negative and became PR negative while pS2 showed a decrease in two cases and an increase in the others.

The remaining six cases were as follows: one case was ER/PR negative and pS2 positive showing no change of ER and PR while pS2 staining increased. One case was ER/pS2 negative and PR positive. ER remained negative while pS2 became positive and PR decreased. Two cases were ER/PR/pS2 negative in pretreatment biopsies. One case showed a positivity of PR in post-treatment tumours and the other a positivity of pS2. Finally two cases were ER positive and PR/pS2 negative. One became ER negative and PR/pS2 positive after treatment. The other showed a decrease in ER positivity, whereas PR and pS2 remained unchanged.

Of these 53 cases, 24 were GSTπ positive and 29 GSTπ negative. Thirty-three showed an increase in immunostaining, whereas 19 showed no change and one a decreased positivity. Relative to c-*erb*B-2, 14 cases were positive and 39 negative. Forty-one cases showed no change while nine cases showed a decreased and three an increased immunostaining.

Lastly, a complete remission was observed in three cases. All were ER positive and showed a decrease of ER staining after tamoxifen. One of three was PR positive/pS2-negative/GSTπ negative/c-*erb*B-2 negative. This phenotype remained unchanged after treatment. Another was PR negative/pS2 positive/GSTπ negative/c-*erb*B-2 positive before treatment and showed no changes except a slight decrease (−5) of pS2 staining. The last one was PR/pS2 negative and GSTπ/c-*erb*B-2 positive and showed an increase of immunostaining for PR, pS2 and GSTπ, whereas c-*erb*B-2 became negative.

### Discussion

Antioestrogens, like other anti-tumour drugs, are suspected to have effects on the expression of various proteins in cancer cells. These are often difficult to evaluate because their study by well-known reference techniques, such as biochemistry, radioimmunology or molecular biology, requires a relatively large amount of fresh material. On the contrary, immunohistochemistry, with the availability of new and reliable antibodies, is becoming more important through retrospective studies. Using this latter technique, we observed significant changes in protein expression in a group of patients first treated by tamoxifen for 5 months and then operated on. We generally found that under tamoxifen, hormonal receptors decreased while GSTπ and pS2 expression increased and c-*erb*B-2 remained stable. Our data concern a retrospective group of patients with perforce selection biases. However, on comparing a range of criteria between this group and the group of non-operated patients, we failed to reveal the bias. A high percentage of ER-positive tumours was observed in both groups. In fact, most of the patients who entered neoadjuvant tamoxifen therapy had a receptor-positive tumour, otherwise they preferably underwent other therapy.

The reliability of a single biopsy for determining gene expression has been questioned. The problem of the tumour sampling was particularly great as gene expression is frequently heterogeneous throughout the tumour and between primary and metastatic sites (Holdaway *et al.*, 1983). However, in spite of this heterogeneity, Hull *et al.* (1983) and Allegra *et al.* (1980) observed only 3% and 15% of major discordances, that is one assay positive and the other one negative between simultaneous assays, respectively. Moreover, provided that cellularity is sufficient, good agreement is found between corecut biopsies or fine-needle aspirates and surgical specimens with respect to HR status (Mauriac *et al.*, 1981; Katz *et al.*, 1990; Frigo *et al.*, 1995). However, slight variations between core biopsies and surgical specimens could certainly be ascribed to this intratumoral heterogeneity.

Antioestrogens such as tamoxifen, have a tumoristatic rather than a tumoricidal effect on breast cancer cells (Warri *et al.*, 1993; Rochefort *et al.*, 1991). In the cell, they have two sites of action. They chiefly compete with oestrogen to bind on to oestrogen receptors, inducing conformational changes of the receptor (Katzenellenbogen *et al.*, 1985). They additionally have high affinity for microsomial antioestrogen binding sites (AEBS) to which oestrogens do not bind. These AEBS are present in equal concentrations in breast cancer cells whatever the ER status (Katzenellenbogen *et al.*, 1985). Antioestrogens have several molecular effects. They block cells in G0−G1 stage of cell cycle inducing the arrest of cell proliferation (Sutherland *et al.*, 1983). They also down-regulate oestrogen-stimulated secretion of several specific proteins (Horwitz *et al.*, 1978; Kida *et al.*, 1989; Daly and Darbre, 1990; Chalbos *et al.*, 1993; Warri *et al.*, 1993). Some of these effects are reversible by oestradiol (Lippman *et al.*, 1986; Gottardis *et al.*, 1988; Daly and Darbre, 1990). A recent report has suggested that the growth-inhibitory effects of tamoxifen may be explained in part by its ability to disrupt a complex between ER, ERAP160 (an ER-associated protein supposed to mediate oestradiol dependent transcriptional activation) and other factors necessary for transactivation (Halachmi *et al.*, 1994). Consequently, ER-positive (ER+) breast cancer cells are more likely to respond to antioestrogen than ER-negative (ER−) breast cancer cells (Katzenellenbogen *et al.*, 1985). In fact, endocrine therapy affects the proliferation of both ER+ and ER− cells, clones of the human breast cancer cell line MCF-7 (Noguchi *et al.*, 1990). Human breast cancer cells also secrete growth factors. In hormone-dependent cells, several are oestrogen-regulated, whereas in cells which acquire independence they are constitutively increased (Lippman *et al.*, 1986). Furthermore, the antioestrogenic properties and the antigrowth factor effects of antioestrogens can be dissociated, thus indicating that the latter is not a direct consequence of the former (Chalbos *et al.*, 1993). Additionally, antioestrogens such as tamoxifen, behave as a partial agonist−antagonist, depending on the target tissue (Gottardis *et al.*, 1988) and on the nature of the gene (Berry *et al.*, 1990). Moreover, it has dual oestrogenic/antioestrogenic properties, being dose dependent (Horwitz *et al.*, 1978) and time-dependent (Waseda *et al.*, 1981; Melchor *et al.*, 1990; Vering *et al.*, 1993). At lower doses or short-term administration (1−2 weeks) tamoxifen may be oestrogenic whereas at higher levels or long-term administration (>3−4 weeks) the antioestrogenic properties are observed.

*In vitro* and *in vivo* studies in breast cancer cells have shown, in accordance with our IHC results, a decrease of ER content following long-term antioestrogen therapy (>3 weeks) (Allegra *et al.*, 1980; Waseda *et al.*, 1981; Taylor *et al.*, 1982; Holdaway *et al.*, 1983; Melchor *et al.*, 1990; Noguchi *et al.*, 1990). Likewise 78% of our tumours evaluated by the DCC assay after tamoxifen therapy showed a decrease in ER content. It is not excluded that tamoxifen occupying ER sites may result in artificially low ER measurements by DCC (Hull *et al.*, 1983), but similar results are obtained with different techniques such as IHC, DCC and hydroxylapatite assays. A false negativity of the

IHC assay (interference of tamoxifen with 1D5 paraffin assay), although not excluded, will imply complete masking of antigenic epitopes. As demonstrated by Taylor *et al.*, the fall in ER content measured by the DCC assay, could also be related to reduced cellularity of the specimen. This was true in responding tumours. But this change was also observed in non-responding tumours where cellularity was infrequently reduced (Taylor *et al.*, 1982). Using the IHC technique, the cellularity of the specimen, provided that sufficient material was examined to be representative of the tumour, could not be responsible for the change of ER content since results are done in terms of percentage of tumour cells. In our group of progressive disease, three of five tumours showed pretreatment high ER-positivity and were completely negative after treatment with a high post-treatment cellularity. Excluding these possibilities of false negativity, molecular effects of tamoxifen should be considered. Paradoxically, antioestrogens do not prevent oestrogen receptor synthesis nor do they accelerate or block ER degradation in MCF-7 cells (Katzenellenbogen *et al.*, 1985). As already noted by Allegra *et al.* (1980), this could suggest that hormonal therapy selectively eliminated ER+ cells. So the clear reduction of ER content in tumours under tamoxifen could be at least in part consistent with the disappearance of ER-positive clones and/or the development of ER-negative clones, rather than with the disappearance of ER expression within cells themselves.

This hypothesis is consistent with our findings concerning variations of PR under tamoxifen treatment. We found a significant decrease of PR under tamoxifen, but close analysis of the results showed a more irregular behaviour. Whereas 59% of our tumours lost some or all of their PR expression, 26% showed no variation and 15% an increase. This has previously been observed by other authors in a short series of 14 patients (Melchor *et al.*, 1990). An *in vitro* study showed similar results: following oestrogen deprivation, some breast cancer cell lines and their subclones behave differently, showing a low level of PR (cell line ZR-75-1, clone 4), a high level of PR (ZR-75-1, clone 11-A) or an unchanged level (cell line T47D) (Daly and Darbre, 1990). Tamoxifen is known to down-regulate PR through ER (Horwitz *et al.*, 1978). Thus, it is possible that tamoxifen (partially or completely occupying ER sites) down-regulation of PR through ER was not complete, especially in non-responding tumours. However, in the hypothesis of cloned selection by tamoxifen, either negative or positive PR phenotypes could also be encountered, more especially as cells lose oestrogen receptors and so control of PR expression.

*In vitro*, pS2 expression is induced by oestrogen (Masiakowski *et al.*, 1982; Jakowlew *et al.*, 1984; Kida *et al.*, 1989; Daly and Darbre, 1990) and this effect is reversible either by oestradiol withdrawal or antioestrogen therapy (Kida *et al.*, 1989; Warri *et al.*, 1991, 1993) resulting in a decrease in pS2 level. But antioestrogens alone, i.e. in the absence of oestrogen, have no effect on pS2 level (Kida *et al.*, 1989). So, although we expected a decrease, we noticed on our series of tumours a relative increase of pS2 expression following tamoxifen administration. Indeed, 12 cases showed no variation and 25 cases showed little variation (approximately 10%). Thus, contrary to *in vitro* studies, our results *in vivo* suggest that pS2 regulation depends on other additional non-oestrogenic mechanisms that may be activated by tamoxifen. They could also reflect acquisition or development of a clone with a hormone independent phenotype. Brünner *et al.* (1993) showed that the latter is associated with modifications in the expression of some oestrogen-regulated genes while ER expression itself remains stable. For instance, these modifications were an increase in pS2 mRNA level while the PR level was variable. Under tamoxifen therapy, an increase in the expression of some growth factors has also been reported. For example, transforming growth factor (TGF-$\beta$) has a growth-inhibitory effect and is stimulated by antioestrogen (Lippman *et al.*, 1986; Daly and Darbre, 1990). The pS2 protein is suspected to have a growth factor function (Rio *et al.*, 1988; Jakowlew *et al.*, 1984). However, it is not involved in the growth-stimulatory effect of oestrogen (Kida *et al.*, 1989). If its increase reflects acquisition of the hormone independent phenotype, we should find a relationship with the response to endocrine therapy. We failed to demonstrate any significant relationship, which suggests that the mechanisms of resistance are complex.

A significant increase in GST$\pi$ expression was observed following tamoxifen treatment. GST$\pi$ gene is highly expressed in ER-negative breast cancer cell lines (Morrow *et al.*, 1992) and tumours (Howie *et al.*, 1989; Moscow *et al.*, 1988; Gilbert *et al.*, 1993). Comparing $ER^-$ and $ER^+$ cell lines, Morrow *et al.* (1992) showed that endogenous GST$\pi$ gene transcription rates are similar in both cell lines but the stability of endogeneous GST$\pi$ mRNA is extraordinarily higher in ER-negative cells. Apart from a direct or indirect effect of tamoxifen on gene regulation, this possible post-transcriptional mechanism could explain our results. As tumours gain in ER-negative cells, they gain in GST$\pi$ expression by increased stability of mRNA.

In the ER+ T47D and ZR-75-1 cell lines (Dati *et al.*, 1990; Warri *et al.*, 1991; Le Roy *et al.*, 1991) oestrogens down-regulated the expression of c-*erb*B-2 and this effect could be reversed by antioestrogens (Read *et al.*, 1990; Warri *et al.*, 1991). On the other hand, no effect of oestradiol on c-*erb*B-2 RNA could be observed in ER− cell line (Le Roy *et al.*, 1991). Moreover Le Roy *et al.* (1991) and Warri *et al.* (1991) failed to demonstrate an effect of antioestrogens on c-*erb*B-2 expression in breast cancer cell lines grown in a steroid-deprived medium (without oestrogen). *In vivo* studies showed conflicting results. In nude mice (Warri *et al.*, 1991) tamoxifen treatment was associated with enhanced expression of c-*erb*B2 and growth arrest. This is surprising since amplification and overexpression of c-*erb*B-2 usually correlate with poor prognosis and increased growth rate (Tsuda *et al.*, 1990; May *et al.*, 1990; Toikkanen *et al.*, 1992). In contrast, the studies of Le Roy *et al.* (1991) showed lower c-*erb*B-2 RNA levels in a tamoxifen-treated group of patients in comparison with an untreated group, but only in a subset of ER-negative tumours, whereas there was no difference in the $ER^+$ group. In our study, no significant variations of c-*erb*B-2 were observed under tamoxifen therapy. However, only 19 of 74 tumours were initially c-*erb*B-2 positive. In this subset with initially 80% of $ER^+$ tumours, we observed a decrease under tamoxifen close to significance ($P = 0.07$). Our results are more consistent with the data of Le Roy *et al.* (1991) and with the development of an ER-negative clone.

A study of tamoxifen effects, *in vivo*, on breast cancer cells should lead to a better understanding of antioestrogens' mechanism of action. It should lead to the definition of hormone-sensitive and resistant criteria. Althoug no definite relationship was demonstrated between marker variations and response to endocrine therapy, we believe that modifications observed under tamoxifen therapy favour clonal selection. Further analyses are needed to address this point in more detail.

**Acknowledgements**
We would like to thank G Siérankowski and J F Déridet for technical assistance, V Picot for helping in statistical analysis and I Le Pollès for the typing of the manuscript.

### References

ALLEGRA JC, BARLOCK A, HUFF KK AND LIPPMAN ME. (1980). Changes in multiple or sequential oestrogen receptor determinations in breast cancer. *Cancer*, **45**, 792–794.

BERRY M, METZGER D AND CHAMBON P. (1990). Role of two activating domains of the estrogen receptor in cell-type and promoter context-dependent agonistic activity of the antioestrogen 4-hydroxytamoxifen. *EMBO J.*, **9**, 2811–2818.

BRUNNER N, BOULAY V, FOJO A, FRETER CE, LIPPMAN ME AND CLARKE R. (1993). Acquisition of hormone-independent growth in MCF-7 cells is accompanied by increased expression of oestrogen-regulated genes but without detectable DNA amplifications. *Cancer Res.*, **53**, 283–290.

CHALBOS D, PHILIPS A, GALTIER F AND ROCHEFORTH H. (1993). Synthetic antioestrogens modulate induction of pS2 and cathepsin-D messenger ribonucleic acid by growth factors and adenosine 3′, 5′-monophosphate in MCF-7 cells. *Endocrinology*, **133**, 571–576.

DALY RJ AND DARBRE PD. (1990). Cellular and molecular events in loss of oestrogen sensitivity in ZR-75-1 and T-47-D human breast cancer cells. *Cancer Res.*, **50**, 5868–5875.

DATI C, ANTONIOTTI S, TAVERNA D, PERROTEAU I AND DE BORTOLI M. (1990). Inhibition of c-*erb*B-2-oncogene expression by oestrogens in human breast cancer cells. *Oncogene*, **5**, 1001–1006.

DORION-BONNET F, QUENEL N, COINDRE JM, MAURIAC L, BONICHON F, DURAND M, WAFFLARD J, MOSCOW JA, COWAN KH AND GUALDE N. (1993). Expression of the GST$\pi$ gene and response to tamoxifen therapy in locally advanced breast carcinomas. *Ann. NY Acad. Sci.*, **68**, 182–185.

EARLY BREAST CANCER TRIALISTS COLLABORATIVE GROUP. (1992). Systemic treatment of early breast cancer by hormonal, cytotoxic, or immune therapy. 133 randomised trials involving 31 000 recurrences and 24 000 deaths among 75 000 women. *Lancet*, **339**, 1–15, 71–85.

FRIGO B, PILOTTI S, ZURRIDA S, ERMELLINO L, MANZARI A AND RILKE F. (1995). Analysis of estorgen and progesterone receptors on preoperative fine-needle aspirates. *Breast Cancer Res. Treat.*, **33**, 179–184.

GILBERT L, ELWOOD LJ, MERINO M, MASOOD S, BARNES R, STEINBERG SM, LAZAROUS DF, PIERCE L, D'ANGELO T, MOSCOW JA, TOWNSEND AJ AND COWAN KH. (1993). A pilot study of Pi-class glutathione S-transferase expression in breast cancer: correlation with oestrogen receptor expression and prognosis in node-negative breast cancer. *J. Clin. Oncol.*, **11**, 49–58.

GOTTARDIS MM, ROBINSON SP, SATYASWAROOP PG AND JORDAN VG. (1988). Contrasting actions of tamoxifen on endometrial and breast tumor growth in the athymic mouse. *Cancer Res.*, **48**, 812–815.

HALACHMI S, MARDEN E, MARTIN G, MACKAY H, ABBONDANZA C AND BROWN M. (1994). Oestrogen receptor-associated proteins: possible mediators of hormone-induced transcription. *Science*, **264**, 1455–1458.

HENRY JA, NICHOLSON S, HENNESY C, LENNARD TWJ, MAY FEB AND WESTLEY BR. (1989). Expression of the oestrogen regulated pNR-2 mRNA in human breast cancer: relation to oestrogen receptor mRNA levels and response to tamoxifen therapy. *Br. J. Cancer*, **61**, 32–38.

HENRY JA, PIGGOTT NH, MALLICK UK, NICHOLSON S, FARNDON JR, WESTLEY BR AND MAY FEB. (1991). pNR-2/pS2 immunohistochemical staining in breast cancer: correlation with prognostic factors and endocrine response. *Br. J. Cancer.*, **63**, 615–622.

HOLDAWAY IM, FRACP MD AND BOWDITCH JV. (1983). Variation in receptor status between primary and metastatic breast cancer. *Cancer*, **52**, 479–485.

HORWITZ KB, KOSEKI Y AND McGUIRE WL. (1978). Oestrogen control of progesterone receptor in human breast cancer: role of estradiol and antioestrogen. *Endocrinology*, **103**, 1742–1751.

HOWIE AF, MILLER WR, HAWKINS RA, HUTCHINSON AR AND BECKETT GJ. (1989). Expression of glutathione S-transferase B1, B2, Mu and Pi in breast cancers and their relationship to oestrogen receptor status. *Br. J. Cancer*, **60**, 834–837.

HULL III DF, CLARK GM, OSBORNE K, CHAMNESS C, KNIGHT III WA AND McGUIRE WL. (1983). Multiple oestrogen receptor assays in human breast cancer. *Cancer Res.*, **43**, 413–416.

HURLIMANN J, GEBHARD S AND GOMEZ F. (1993). Oestrogen receptor, progesterone receptor, pS2, ERD5, HSP27 and cathepsin D in invasive ductal breast carcinomas. *Histopathology*, **23**, 239–248.

JAKOWLEW SB, BREATHNACH R, JELTSCH JM, MASIAKOWSKI P AND CHAMBON P. (1984). Sequence of the pS2 mRNA induced by oestrogen in the human breast cancer cell line MCF-7. *Nucleic Acids Res.*, **12**, 2861–2878.

KATZ RL, PATEL S, SNEIGE N, FRITSCHE Jr, HORTOBAGYI GN, AMES FC, BROOKS T AND ORDONEZ NG. (1990). Comparison of immunocytochemical and biochemical assays for oestrogen receptor in fine needle aspirates and histologic sections from breast carcinomas. *Breast Cancer Res. Treat.*, **15**, 191–203.

KATZENELLENBOGEN BS, MILLER MA, MULLICK A AND SHEEN YY. (1985). Antioestrogen action in breast cancer cells: modulation of proliferation and protein synthesis, and interaction with oestrogen receptors and additional antioestrogen binding sites. *Breast Cancer Res. Treat.*, **5**, 231–243.

KIDA N, YOSHIMURA T, MORI K AND HAYASHI K. (1989). Hormonal regulation of synthesis and secretion of pS2 protein relevant growth of human breast cancer cells (MCF-7). *Cancer Res.*, **49**, 3494–3498.

LE ROY X, EXCOT C, BROUILLET JP, THEILLET C, MAUDELONDE T, SIMONY-LAFONTAINE J, PUJOL H AND ROCHEFORT H. (1991). Decrease of c-*erb*B-2 and c-myc RNA levels in tamoxifen-treated breast cancer. *Oncogene*, **6**, 431–437.

LIPPMAN ME, DICKSON RB, BATES S, KNABBE C, HUFF K, SWAIN S, MCMANAWAY M, BRONZERT D, KASID A AND GELMANN EP. (1986). Autocrine and paracrine growth regulation of human breast cancer. *Breast Cancer Res. Treat.*, **7**, 59–70.

de MASCAREL I, SOUBEYRAN I, MAC GROGAN G, WAFFLART J, BONICHON F, DURAND M, AVRIL A, MAURIAC L, TROJANI M AND COINDRE JM. (1996). Immunohistochemical analysis of oestrogen receptors in 938 breast carcinomas: concordance with biochemical assay and prognostic significance. *App. Immunohistochem.* In press.

MASIAKOWSKI P, BREATHNACH R, BLOCH J, GANNON R, KRUST A AND CHAMBON P. (1982). Cloning of cDNA sequences of hormone-regulated genes from the MCF-7 human breast cancer cell line. *Nucleic Acids Res.*, **10**, 7895–7903.

MAURIAC L, WAFFLART J, DURAND M, PARSI B, de MASCAREL I, TROJANI M AND MEUGE-MORAW C. (1981). Contribution of drill-biopsies to pre-treatment investigation of breast adenocarcinomas. *Bull. Cancer.*, **68**, 417–421.

MAY E, MOURIESSE H, MAY-LEVIN F, QIAN JF, MAY P AND DELARUE JC. (1990). Human breast cancer: identification of populations with a high risk of early relapse in relation to both oestrogen receptor status and c-*erb*B-2 overexpression. *Br. J. Cancer*, **62**, 430–435.

MELCHOR JC, RODRIGUEZ-ESCUDERO FJ, LUJAN S AND CORCOSTEGUI B. (1990). Variation of oestrogen and progesterone receptor status in breast cancer after tamoxifen therapy. *Oncology*, **47**, 467–470.

MORROW CS, CHIU J AND COWAN KH. (1992). Posttranscriptional control of glutathione S-transferase $\pi$ gene expression in human breast cancer cells. *J. Biol. Chem.*, **267**, 10544–10550.

MOSCOW JA, TOWNSEND AJ AND GOLDSMITH ME. *et al.* (1988). Isolation of the human anionic glutathione-S-transferase gene and the relation of its expression to oestrogen receptor content in primary breast cancer. *Proc. Natl. Acad. Sci.*, **85**, 6518–6522.

NICHOLSON RI, McCLELLAND RA, FINLAY P, EATON CL, GULLICK WJ, DIXON AR, ROBERTSON JFR, ELLIS IO AND BLAMEY RW. (1993). Relationship between EGF-R, c-*erb*B-2 protein expression and Ki67 immunostaining in breast cancer and hormone sensitivity. *Eur. J. Cancer.*, **29A**, 1018–1023.

NOGUCHI M, TAJIRI K, TANIYA T, KUMAKI T, ASHIKARI A AND MIYAZAKI I. (1990). Influence of hormones on proliferation of ER-positive cells and ER-negative cells of human breast cancer (MCF-7). *Oncology*, **47**, 19–24.

QUÉNEL N, COINDRE JM, WAFFLART J, BONICHON F, de MASCAREL I, TROJANI M, DURAND M AND AVRIL A. (1995). The prognostic value of c-*erb*B-2 in primary breast carcinomas: a study on 942 cases. *Breast Cancer Res. Treat.*, **35**, 283–291.

READ LD, KEITH D, SLAMON DJ AND KATZELLENBOGEN BS. (1990). Hormonal modulation of HER-2/neu proto-oncogene messenger ribonucleic acid and p185 protein expression in human breast cancer cell lines. *Cancer Res.*, **50**, 3947–3951.

RILKE F, COLNAGHI MI, CASCINELLI N, ANDREOLA S, BALDINI MT, BUFALINO R, DELLA PORTA G, MENARD S, PIEROTTI MA AND TESTORI A. (1991). Prognostic significance of HER-2/neu expression in breast cancer and its relationship to other prognostic factors. *Int. J. Cancer*, **49**, 44–49.

RIO MC, BELLOCQ JP, DANIEL JY, TOMASSETTO C, LATHE R, CHENARD MP, BATZENSCHLAGER A AND CHAMBON P. (1988). Breast cancer-associated pS2 protein: synthesis and secretion by normal stomach mucosa. *Science*, **241,** 705–708.

ROCHEFORT H. (1991). Mechanism of action of high-affinity antioestrogens. *Am. J. Clin. Oncol.*, **14,** S1–S4.

SCHWARTZ LH, KOERNER FC, EDGERTON SM, SAWICKA JM, RIO MC, BELLOCQ JP, CHAMBON P AND THOR AD. (1991). pS2 expression and response to hormonal therapy in patients with advanced breast cancer. *Cancer Res.*, **51,** 624–628.

SOUBEYRAN I, COINDRE JM, WAFFLART J, BONICHON F, de MASCAREL I, TROJANI M, DURAND M AND AVRIL A. (1995). Immunohistochemical determination of pS2 in invasive breast carcinomas: a study on 942 cases. *Breast Cancer Res. Treat.*, **34,** 119–128.

SOUBEYRAN I, QUÉNEL N, COINDRE JM, BONICHON F, DURAND M, WAFFLART J AND MAURIAC L. PS2 protein: a marker improving prediction of response to neoadjuvant tamoxifen in post-menopausal breast cancer patients. Submitted to *Br. J. Cancer*.

SUTHERLAND RL, GREEN MD, HALL RE, REDDEL RR AND TAYLOR IW. (1983). Tamoxifen induces accumulation of MCF-7 human mammary carcinoma cells in the G0–G1 phase of the cell cycle. *Eur. J. Cancer Clin. Oncol.*, **19,** 615–621.

TAYLOR RE, POWLES TJ, HUMPHREYS, J, BETTELHEIM R, DOWSETT M, CASEY AJ, NEVILLE AM AND COOMBES RC. (1982). Effects of endocrine therapy on steroid-receptor content of breast cancer. *Br. J. Cancer*, **45,** 80–85.

TOIKKANEN S, HELIN H, ISOLA J AND JOENSUU H. (1992). Prognostic significance of HER-2 oncoprotein expression in breast cancer: a 30-year follow-up. *J. Clin. Oncol.*, **10,** 1044–1048.

TSUDA H, HIROHASHI S, SHIMOSATO Y, HIROTA T, TSUGANE S, WATANABE S, TERADA M AND YAMAMOTO H. (1990). Correlation between histologic grade of malignancy and copy number of c-*erb*B-2 gene in breast carcinoma. A retrospective analysis of 176 cases. *Cancer*, **65,** 1794–1800.

VERING A, VOCKEL A, STEGMÜLLER M AND BENDER HG. (19[illegible]3). Immunobiochemical assay for determination of nuclear steroid receptors during tamoxifen therapy. *J. Cancer Res. Clin. Oncol.*, **119,** 415–420.

WÄRRI AM, LAINE AM, MAJASUO KE, ALITALO KK AND HÄRKÖNEN PL. (1991). Oestrogen suppression of *erb*B-2 expression is associated with increased growth rate of ZR-75-1 human breast cancer cells *in vitro* and in nude mice. *Int. J. Cancer*, **49,** 616–623.

WÄRRI AM, HUOVINEN RL, LAINE AM, MARTIKAINEN PM AND HÄRKÖNEN PL. (1993). Apoptosis in toremifene-induced growth inhibition of human breast cancer cells *in vivo* and *in vitro*. *J. Natl. Cancer Inst.*, **85,** 1412–1418.

WASEDA N, KATO Y, IMURA H AND KURATA M. (1981). Effects of tamoxifen on oestrogen and progesterone receptos in human breast cancer. *Cancer Res.*, **41,** 1984–1988.

WILSON YG, RHODES M, IBRAHIM NBN, PADFIELD CJH AND CAWTHORN SJ. (1994). Immunocytochemical staining of pS2 protein in fine-needle aspirate from breast cancer is an accurate guide to response to tamoxifen in patients aged over 70 years. *Br. J. Surg.*, **81,** 1155–1158.

WRIGHT C, NICHOLSON S, ANGUS B, SAINSBURY JRC, FARNDON J, CAIRNS J, HARRIS AL AND HORNE CHW. (1992). Relationship between c-erbB-2 protein product expression and response to endocrine therapy in advanced breast cancer. *Br. J. Cancer.*, **65,** 118–121.